# Neutrophil gelatinase-associated lipocalin elimination by renal replacement therapy: minding the membrane!

**DOI:** 10.1186/s13054-016-1258-9

**Published:** 2016-04-05

**Authors:** Patrick M. Honore, Herbert D. Spapen

**Affiliations:** Intensive Care Unit Department, Universitair Ziekenhuis Brussel, Vrije Universiteit Brussel (VUB University), 101, Laarbeeklaan, 1090 Jette, Brussels Belgium

The recently published data of Donadio in *Critical Care* provide convincing evidence that neutrophil gelatinase-associated lipocalin (NGAL), an established key biomarker of acute kidney injury (AKI) in the critically ill, can be effectively removed by renal replacement therapy (RRT) [1]. His findings challenge recent work by Schilder et al. [2] and corroborate our concern regarding the potential removal of NGAL during continuous RRT (CRRT) [3]. NGAL is expected to be eliminated by convection because its molecular weight (MW) lies below the 30-kDa MW cutoff point of “classic” dialysis membranes. Schilder et al. found almost no clearance of NGAL during continuous veno-venous hemofiltration despite using a 40-kDa MW (high) cutoff cellulose triacetate filter [2]. Donadio used the same filter in patients undergoing high-flux maintenance hemodialysis (MHD) but applied an ultrafiltration rate more than twice as high as that of Schilder et al. (8474 versus 3700 ml/h per 100 mm Hg) and a higher sieving coefficient for middle molecules [4]. Moreover, Donadio observed that hemodiafiltration (HDF) largely outperformed high-flux MHD in plasma NGAL removal (52.1 % versus 26.7 % reduction ratio) [4]. This could be explained by a difference in membrane type. Cellulose triacetate filters have very poor adsorption capacity [5]. In contrast, the acrylonitrile (AN) and natrium metallylsulfone copolymer membrane used for MHD in the Donadio study has characteristics similar to those of the highly adsorptive AN69 surface-treated membrane used for CRRT in critically ill patients [5]. As this filter displays a lower MW cutoff than that of cellulose triacetate (30 versus 40 kDa), the remarkable removal of NGAL from plasma in patients undergoing HDF is not explained by increased convection alone but is likely related to additional membrane adsorption.

Taken together, the recent work [4] and comments including the additional data [1] of Donadio underscore that NGAL may lose significance to reflect severity and prognosis of AKI in patients receiving RRT because intensified convection or substantial adsorption (or both) on currently used dialysis membranes enhances plasma clearance of this biomarker.

## Abbreviations

AKI: Acute kidney injury
AN: Acrylonitrile
CRRT: Continuous renal replacement therapy
HDF: Hemodiafiltration
MHD: Maintenance hemodialysis
MW: Molecular weight
NGAL: Neutrophil gelatinase-associated lipocalin
RRT: Renal replacement therapy
